# Engineering a CRISPRoff Platform to Modulate Expression of Myeloid Cell Leukemia (MCL-1) in Committed Oligodendrocyte Neural Precursor Cells

**DOI:** 10.21769/BioProtoc.4913

**Published:** 2024-01-05

**Authors:** Melanie Gil, Catherine A. Hamann, Jonathan M. Brunger, Vivian Gama

**Affiliations:** 1 Neuroscience Graduate Program, Vanderbilt University, Nashville, TN, USA; 2 Vanderbilt Brain Institute, Vanderbilt University, Nashville, TN, USA; 3 Biomedical Engineering, Vanderbilt University, Nashville, TN, USA; 4 Department of Cell and Developmental Biology, Vanderbilt University, Nashville, TN, USA; 5 Vanderbilt Center for Stem Cell Biology, Vanderbilt University, Nashville, TN, USA

**Keywords:** Oligodendrocytes, Oligodendrocyte precursor cells, CRISPR/Cas, Sleeping Beauty, MCL-1, Pluripotent stem cells

## Abstract

In vitro differentiation of human pluripotent stem cell (hPSC) model systems has furthered our understanding of human development. Techniques used to elucidate gene function during early development have encountered technical challenges, especially when targeting embryonic lethal genes. The introduction of CRISPRoff by Nuñez and collaborators provides an opportunity to heritably silence genes during long-term differentiation. We modified CRISPRoff and sgRNA Sleeping Beauty transposon vectors that depend on tetracycline-controlled transcriptional activation to silence the expression of embryonic lethal genes at different stages of differentiation in a stable manner. We provide instructions on how to generate sgRNA transposon vectors that can be used in combination with our CRISPRoff transposon vector and a stable hPSC line. We validate the use of this tool by silencing MCL-1, an anti-apoptotic protein, which results in pre-implantation embryonic lethality in mice; this protein is necessary for oligodendrocyte and hematopoietic stem cell development and is required for the in vitro survival of hPSCs. In this protocol, we use an adapted version of the differentiation protocol published by Douvaras and Fossati (2015) to generate oligodendrocyte lineage cells from human embryonic stem cells (hESCs). After introduction of the CRISPRoff and sgRNAs transposon vectors in hESCs, we silence *MCL*-1 in committed oligodendrocyte neural precursor cells and describe methods to measure its expression. With the methods described here, users can design sgRNA transposon vectors targeting *MCL*-1 or other essential genes of interest to study human oligodendrocyte development or other differentiation protocols that use hPSC model systems.

Key features

• Generation of an inducible CRISPRoff Sleeping Beauty transposon system.

• Experiments performed in vitro for generation of inducible CRISPRoff pluripotent stem cell line amenable to oligodendrocyte differentiation.

• Strategy to downregulate an essential gene at different stages of oligodendrocyte development.

**Graphical overview**


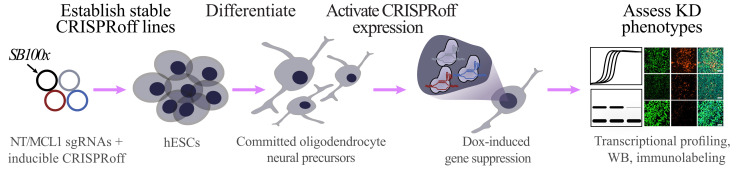


**Workflow for generating inducible CRISPRoff stem cell line and assessing knockdown phenotype in stem cell–derived committed oligodendrocyte neural precursor cells**

## Background

In vitro systems of differentiation have become useful tools to dissect the molecular mechanisms involved in cell fate transitions. The use of human pluripotent stem cells (PSCs) has revolutionized our understanding of early stages of human development that were previously inaccessible. Different gene-targeting techniques, such as RNAi, TALEN, Cas9 nuclease, and CRISPRi/a, are available to investigate the function of specific genes during development [1–3]. Partial gene knockdowns in PSCs are commonly used to study the function of genes that would be lethal if completely knocked out. However, most of these tools have transient genetic modifications and their effectiveness is diminished during in vitro differentiation of PSCs. Nuñez et al. pioneered CRISPRoff, a programmable epigenetic memory writer protein that can heritably silence genes [4]. This tool has greatly benefited the field, allowing developmental researchers to further understand the function of genes during long-term in vitro differentiations, as epigenetic memory is stable. We built on the CRISPRoff system by adding a Sleeping Beauty transposon system containing a reverse tetracycline-controlled transactivator (Figure 1A). Additionally, we designed a single-guide RNA (sgRNA) transposon plasmid that allows for efficient genomic integration (Figure 1B). With the CRISPRoff and sgRNA transposon vectors described here, we were able to develop a novel experimental approach to inducibly silence expression of MCL-1, an anti-apoptotic protein, in oligodendrocyte lineage cells.

Deletion of *Mcl-1* results in pre-implantation embryonic lethality [5]. Consequently, investigating the function of MCL-1 during development has barriers as mouse embryos are not able to implant and PSCs undergo apoptosis once *MCL-1* is knocked down or inhibited via small molecules [6, 7]. This technical barrier has halted further research on the function of MCL-1 during early human neurodevelopment. With CRISPRoff, we silenced expression of *MCL-1* at different stages of neurodevelopment. In this protocol, we outline how this set of tools can be used to investigate the potential function of MCL-1 during development of oligodendrocyte lineage cells. We adapted this technology and linked it to an established oligodendrocyte generation protocol [8] to validate the use of these novel tools during differentiation of human PSCs. Moreover, we detail how to generate sgRNA transposon vectors for other genes of interest that can be adapted into alternative differentiation systems.

There is a lack of literature that combines culturing of human oligodendrocyte lineage cells with epigenetic editing strategies. This protocol aims to bridge that gap by using a proof-of-principle experiment where *MCL-1* is silenced at an early stage of differentiation. This protocol can be used to investigate an array of genes that cause lethality in PSCs or for studying gene function at different stages of oligodendrocyte development.

## Materials and reagents

**Biological materials**

1. H9 human embryonic stem cells (WiCell Research Institute, WA09, NIH Registration Number: 0062)
2. Competent *E. coli* cells (New England Biolabs, catalog number: C2987H/C2987I)
3. pSB-TRE-CRISPRoff-EF1A-TetOn (Addgene, catalog number: 203355)
4. pSB-BbsI-sgRNA (Addgene, catalog number: 203359)
5. pCMV(CAT)T7-SB100 (Addgene, catalog number: 34879)

**Reagents**

1. BbsI-HF endonuclease (New England Biolabs, catalog number: R0539/R0539L)
2. rCutSmart buffer (New England Biolabs, catalog number: B6004S)
3. Quick calf intestinal alkaline phosphatase (CIP) (New England Biolabs, catalog number: M0525S/M0525L)
4. PCR and/or gel extraction purification kit (Invitrogen, catalog number: K220001)
5. T4 DNA ligase reaction buffer (New England Biolabs, catalog number: B0202S)
6. T4 DNA ligase (New England Biolabs, catalog number: M0202S/M0202L)
7. Polynucleotide kinase (PNK) (New England Biolabs, catalog number: M0201S/M0201L)
8. Recommended colony PCR reagents include *Taq* DNA Polymerase with ThermoPol^®^ buffer (New England Biolabs, catalog number: M0267S/M0267L) and deoxynucleotide (dNTP) solution mix (New England Biolabs, catalog number: N0447S/N0447L)
9. Difco^TM^ LB broth, Miller (Luria-Bertani) (Becton Dickinson, catalog number: 244610)
10. LB agar, Miller (Fisher Scientific, catalog number: BP1425-500/BP1425-2)
11. Plasmid DNA Miniprep kit (Qiagen, catalog number: 27104)
12. Ampicillin sodium salt (Thermo Fisher Scientific, catalog number: 611770250/611770050)
13. TRIzol^TM^ reagent (Thermo Fisher Scientific, catalog number: 15596026)
14. RNaseZAP (Thermo Fisher Scientific, catalog number: AM9780)
15. Chloroform (Millipore Sigma, catalog number: C2432)
16. 2-Propanol (Millipore Sigma, catalog number: I9516)
17. Ethyl alcohol (Millipore Sigma, catalog number: 459836)
18. Diethyl pyrocarbonate (DEPC)-treated water (KD Medical, catalog number: RGE3050)
19. Ethylenediaminetetraacetic acid (EDTA), 0.5 M, pH 8.0, molecular biology grade, DEPC-treated (Millipore Sigma, catalog number: 324506)
20. DNase I (New England Biolabs, catalog number: M0303)
21. Thermo Fisher High-Capacity cDNA Reverse Transcription kit (Thermo Fisher Scientific, catalog number: 4368814)
22. SYBR Green PCR Master Mix (Thermo Fisher Scientific, catalog number: 4309155)
23. Nuclease-free water (Thermo Fisher Scientific, catalog number: AM9906)
24. Phosphate-buffered saline (PBS) tablets (Fisher BioReagents, catalog number: BP2944100)
25. Paraformaldehyde (PFA) 16% aqueous solution (Electron Microscope Sciences, catalog number: 15710-S)
26. Triton X-100 (Millipore Sigma, catalog number: T87876)
27. Bovine serum albumin (BSA) (Millipore Sigma, catalog number: A4503-100G)
28. Fluoromount-G (Thermo Fisher Scientific, catalog number: 00-4958-02)
29. Sodium dodecyl sulfate (SDS) (Millipore Sigma, catalog number: L3771)
30. Trizma^®^ base (Tris Base) (Millipore Sigma, catalog number: T6066)
31. Glycine (Millipore Sigma, catalog number: G7126)
32. Sodium chloride (NaCl) (Millipore Sigma, catalog number: S7653)
33. Hydrochloric acid (HCl) (Millipore Sigma, catalog number: 258148)
34. Dry powder milk (RPI, catalog number: M17200)
35. TWEEN^®^ 20 (Millipore Sigma, catalog number: P7949)
36. Phosphatase inhibitor PhosSTOP (Roche, Thermo Fisher Scientific, catalog number: 4906845001)
37. Protease inhibitor phenylmethylsulfonyl fluoride (PMSF) (Roche, Thermo Fisher Scientific, catalog number: 10837091001)
38. cOmplete^TM^, EDTA-free protease inhibitor cocktail (PIC) (Roche, Thermo Fisher Scientific, catalog number: 4693132001)
39. Pierce bicinchoninic acid (BCA) Protein Assay kit (Thermo Fisher Scientific, catalog number: 23227)
40. 4× Blot^TM^ lithium dodecyl sulfate (LDS) sample buffer (Thermo Fisher Scientific, catalog number: B0007)
41. 2-Mercaptoethanol (Bio-Rad, catalog number: 1610710)
42. Hoechst 33342 solution (Thermo Fisher Scientific, catalog number: 62249)
43. 4%–20% Mini-PROTEAN^®^ TGX^TM^ Precast protein gels (Bio-Rad catalog number: 4561091)
44. mTeSR1 (STEMCELL Technologies, catalog number: 85850)
45. Matrigel Matrix hESC-qualified mouse (Corning, catalog number: 354277)
46. DMEM/F-12 (N-2-hydroxyethylpiperazine-N-2-ethane sulfonic acid) HEPES (Thermo Fisher Scientific, catalog number: 11330032)
47. Gentle cell dissociation reagent (STEMCELL Technologies, catalog number: 100-0485)
48. ROCK1 and ROCK2 inhibitor Y-27632 (STEMCELL Technologies, catalog number: 72307)
49. Opti-MEM I reduced serum medium (Thermo Fisher Scientific, catalog number: 31985070)
50. TransIT-LT1 transfection reagent (Mirus, catalog number: 2304)
51. Accutase (Innovative Cell Technologies, catalog number: AT104)
52. Puromycin dihydrochloride from *Streptomyces alboniger* (Millipore Sigma, catalog number: P8833-10MG)
53. Hygromycin B (Thermo Fisher Scientific, catalog number: 10687010)
54. Caspase inhibitor Quinoline-Val-Asp-Difluorophenoxymethylketone (Q-VD-Oph) (SM Biochemicals LLC, catalog number: SMP001-5MG)
55. Doxycycline hyclate (Tocris Bioscience, catalog number: 4090)
56. Insulin solution human (Thermo Fisher Scientific, catalog number: I9278)
57. DMEM/F-12 (Thermo Fisher Scientific, catalog number: 11320033)
58. Penicillin-Streptomycin (Thermo Fisher Scientific, catalog number: 15140122)
59. MEM non-essential amino acids solution 100× (Thermo Fisher Scientific, catalog number: 11140050)
60. GlutaMAX supplement (Thermo Fisher Scientific, catalog number: 35050061)
61. Retinoic acid (Millipore Sigma, catalog number: R2625)
62. TGF-beta/Smad inhibitor LDN-193189 (Reprocell, catalog number: 04-0074)
63. TGF-beta/Smad inhibitor Stemolecule SB431542 (Reprocell, catalog number: 04-0010-10)
64. N-2 supplement 100× (Thermo Fisher Scientific, catalog number: 17502048)
65. Smoothened agonist (SAG) (STEMCELL Technologies, catalog number: 73412)
66. Ultrapure DNase/RNase-free distilled water (Thermo Fisher Scientific, catalog number: 10977015)

**Solutions**

1. DNase I reaction (see Recipes)
2. Reverse transcription master mix (see Recipes)
3. 1% Triton (see Recipes)
4. 4% PFA (see Recipes)
5. 10% BSA (see Recipes)
6. Lysis buffer (see Recipes)
7. 10× Tris-Gly-SDS buffer (see Recipes)
8. 20× TBS (see Recipes)
9. TBST (see Recipes)
10. 5% Milk in TBST (see Recipes)
11. Basal medium (see Recipes)
12. Neural induction medium (NIM) (see Recipes)
13. N2 medium (see Recipes)

**Recipes**

1. **DNase I reaction**
*Note: Keep on ice and use immediately after preparation.*
  **Table:** 

Reagent	Final concentration	Quantity or Volume
DNase I reaction buffer (10×)	1×	1 µL
DNase I (RNase-free)	n/a	0.2 µL
RNA sample	n/a	2 µg
Nuclease-free H_2_O	n/a	Up to 10 µL
Total	n/a	10 µL
2. **Reverse transcription master mix**
*Note: Keep on ice and use immediately after preparation.*
  **Table:** 

Reagent	Final concentration	Quantity or Volume
10× RT buffer	1×	2 µL
25× dNTP mix	1×	0.8 µL
10× RT random primers	1×	2 µL
MultiScribe^TM^ reverse transcriptase	n/a	Up to 10 µL
Nuclease-free H_2_O	n/a	4.2 µL
Total	n/a	10 µL
3. **1% Triton**
*Note: Store at 4 °C.*
  **Table:** 

Reagent	Final concentration	Quantity or Volume
Triton X-100	1%	50 µL
1× PBS	n/a	49.5 mL
4. **4% PFA**
  **Table:** 

Reagent	Final concentration	Quantity or Volume
16% PFA	4%	12.5 mL
1× PBS	n/a	37.5 mL
5. **10% BSA**
*Note: Store at 4 °C.*
  **Table:** 

Reagent	Final concentration	Quantity or Volume
BSA	10%	5 g
1× PBS	n/a	50 mL
6. **Lysis buffer**
*Note: Keep on ice and use immediately after preparation.*
  **Table:** 

Reagent	Final concentration	Quantity or Volume
1% Triton (Recipe 3)	0.79%	79 µL
10× PhosSTOP	1×	10 µL
10× PIC	1×	10 µL
100 mM PMSF	1 mM	1 µL
Total	n/a	100 µL
7. **10× Tris-Gly-SDS buffer**
  **Table:** 

Reagent	Final concentration	Quantity or Volume
Tris Base	n/a	121.1 g
Glycine	n/a	576 g
SDS	1%	200 mL
ddH_2_O	n/a	Up to 4 L
Total	n/a	4 L
8. **20× TBS**
*Note: Adjust pH to 7.6 with HCl.*
  **Table:** 

Reagent	Final concentration	Quantity or Volume
Tris Base	n/a	193.6 g
NaCl	n/a	640 g
ddH_2_O	n/a	Up to 4 L
Total	n/a	4 L
9. **TBST**
  **Table:** 

Reagent	Final concentration	Quantity or Volume
20× TBS	1×	50 mL
ddH_2_O	n/a	949 mL
TWEEN^®^ 20	0.1%	1 mL
Total	n/a	1 L
10. **5% Milk in TBST**
  **Table:** 

Reagent	Final concentration	Quantity or Volume
Dry powder milk	5%	5 g
TBST	n/a	100 mL
Total	n/a	100 mL
11. **Basal medium**
*Note: Filter sterilize and store at 4 °C.*
  **Table:** 

Reagent	Final concentration	Quantity or Volume
DMEM/F12	n/a	485 mL
MEM non-essential amino acids solution	1×	5 mL
GlutaMAX supplement	1×	5 mL
Penicillin-Streptomycin	1×	5 mL
2-Mercaptoethanol	55 µM	1.93 µL
Total	n/a	500 mL
12. **Neural induction medium (NIM)**
Note: Store at 4 °C. *Add small molecules fresh daily.
  **Table:** 

Reagent	Final concentration	Quantity or Volume
Basal medium (Recipe 7)	n/a	50 mL
Insulin	25 µg/mL	108 µL
SB431542*	10 µM	25 µL
LDN193189*	250 nM	6.25 µL
Retinoic acid*	100 nM	50 µL
Total	n/a	50 mL
13. **N2 medium**
Note: Store at 4 °C. *Add small molecules fresh daily.
  **Table:** 

Reagent	Final concentration	Quantity or Volume
Basal medium (Recipe 7)	n/a	49.5 mL
N-2 supplement 100×	1×	500 µL
SAG*	250 nM	5 µL
RA*	100 nM	50 µL
Total	n/a	50 mL

**Laboratory supplies**

1. 1.5 mL microcentrifuge tubes (Fisherbrand, catalog number: 05-408-1317)
2. PCR tubes (Thermo Fisher Scientific, catalog number: AB2000)
3. Costar 6-well clear TC-treated well plates (Corning, catalog number: 3516)
4. Cell lifter (Corning, catalog number: 3008)
5. Surface-treated sterile tissue culture plates (Fisherbrand, catalog number: FB012928)
6. Stericup-GP 500 mL Express Plus (Millipore Sigma, catalog number: S2GPU05RE)
7. 35 mm glass-bottom dish with 14 mm micro-well #1.5 cover glass (Cellvis, catalog number: D35-14-1.5-N)

## Equipment

1. Applied Biosystems^TM^ QuantStudio^TM^ 3 Real-Time PCR System, 96-well, 0.2 mL, laptop (Thermo Fisher Scientific, catalog number: A28567)
2. Thermocycler (Thermo Fisher Scientific, catalog number: 4484073)
3. Incubated shaker (Thermo Fisher Scientific, catalog number: SHKE6000)
4. Synergy HT Microtiter Plate reader (BioTek, catalog number: 7091000)
5. Amersham Imager 600 (General Electric, catalog number: AI600)
6. Stirring Hotplates (Thermo Fisher Scientific, catalog number: SP88854100)
7. Mini-PROTEAN Tetra Vertical Electrophoresis Cell (Bio-Rad, catalog number: 1658004
8. PowerPac^TM^ HC power supply (Bio-Rad, catalog number: 1645052)
9. Spinning disk confocal microscope (Nikon Eclipse Ti-E equipped with a Plan Apo Lambda 20× 0.75 NA WD 1.00 mm objective and an Andor DU-897 EMCCD camera)

## Software and datasets

1. Prism v9 (GraphPad, 10/24/2020)
2. Image Studio Lite
3. SnapGene v6.2.1

## Procedure

- **Cloning strategy**
  Sleeping Beauty transposon plasmids (Figure 1) were cloned using NEBuilder^®^ HiFi DNA Assembly Mastermix and are available from Addgene as noted in section *Biological materials* above.
  **Figure 1.**
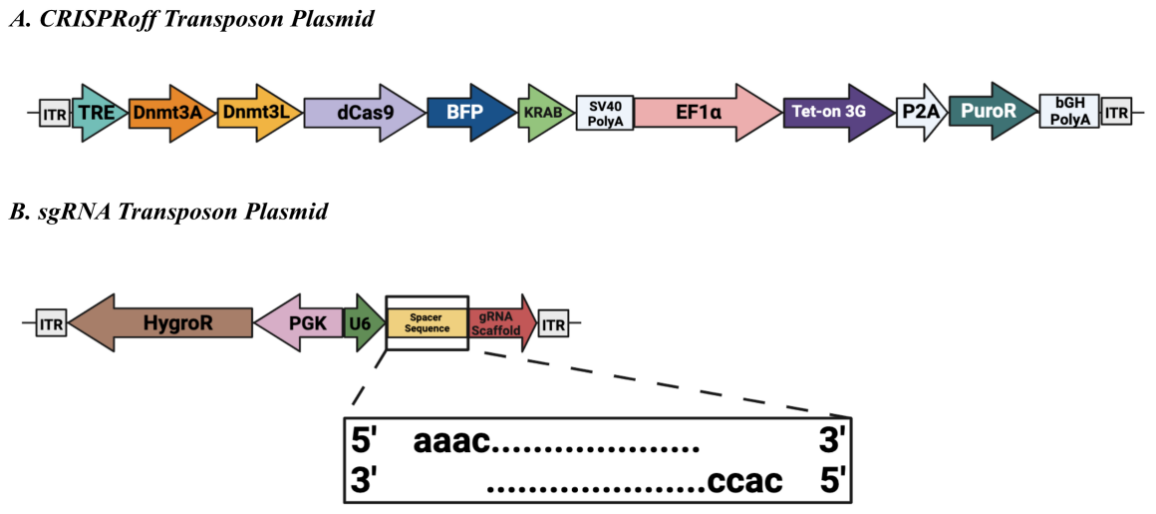
Sleeping Beauty transposon plasmids. (A) CRISPRoff transposon plasmid (Addgene #203355) encodes constitutive expression of the puromycin N-acetyl-transferase gene (PuroR) and a reverse tetracycline-controlled transactivator (Tet-on 3G) that enables doxycycline-dependent activation of the tetracycline response element (TRE)-driven transgene. The TRE regulates transcription of the CRISPRoff enzyme, composed of the DNA methyltransferases 3a (Dnmt3a) and 3-like (Dnmt3L, as well as dCas9-KRAB fused to blue fluorescent protein (BFP). SV40 PolyA: Simian virus 40 polyadenylation signal sequence. EF1α: Elongation factor-1 alpha. P2A: Porcine teschovirus-1 2A self-cleaving peptide. bGH PolyA: Bovine growth hormone polyadenylation signal sequence. (B) Single-guide RNA (sgRNA) transposon plasmid (Addgene #203359) encodes constitutive expression of the hygromycin phosphotransferase (HygroR) transgene. The U6 type III polymerase promoter constitutively drives expression of the sgRNA, which consists of a constant scaffold region and a user-defined spacer sequence. Inset: Annealed sense and anti-sense oligos should include the appropriate BbsI overhangs for successful ligation. Both transposon plasmids contain inverted terminal repeats (ITRs) spanning either end for successful genomic integration mediated by the Sleeping Beauty 100× transposase.
- **sgRNA design**
  Numerous resources are available to aid in the selection of target-specific sgRNA sequences. For example, Horlbeck et al. and Sanson et al. have established sgRNA design rules for suppressing transcripts in the human genome via CRISPRi [9, 10]. Horlbeck et al. generated a database of predicted, active sgRNAs against the human genome, and this database was used to design sgRNAs in the seminal CRISPRoff work, suggesting that CRISPRi libraries serve as a reasonable starting point for selecting sgRNAs for CRISPRoff. The CRISPRi library developed by Sanson et al. is available via the CRISPick web portal located at https://portals.broadinstitute.org/gppx/crispick/public. Users select the appropriate genome editing mechanism (i.e., CRISPRi), and Cas protein (i.e., SpyoCas9) to identify sgRNAs with predicted high and specific activity. Addgene also maintains a table of gRNA sequences available from the depository, which users can mine to determine whether genes of interest have been effectively targeted in CRISPRi or CRISPRoff experiments (https://www.addgene.org/crispr/grnas/). It is useful to note, however, that effective sgRNAs for CRISPRi are typically localized to a narrow window downstream of the transcription start site (TSS), while CRISPRoff-mediated gene repression displays a wide targeting window spanning a distance in excess of 2 kb from the TSS. Thus, candidate sgRNA sequences that are not predicted by tools referenced above may be empirically tested and validated. Critically, users will need to identify potential Cas9 target sites that contain a protospacer adjacent motif (PAM) for Spyo Cas9 (5′-NGG-3′). These sites can be identified using tools such as the UCSC Genome Browser (https://genome.ucsc.edu/). The sgRNA sequences used in this protocol can be found in Table S1.
- **sgRNA transposon plasmid design**
  1. Preparation of sgRNA backbone
    - Perform BbsI digest and backbone dephosphorylation by combining ~1 µg of sgRNA transposon plasmid (Addgene #203359), 1 µL of BbsI endonuclease, 2 µL of rCutSmart Buffer, 1 µL of Quick CIP, and water to bring to a total final volume of 20 µL in a sterile tube. Place at 37 °C for 1–3 h.
    - Perform PCR purification or gel extraction to purify the digested backbone. In our experience, a PCR purification is adequate as the excised fragment is 22 bp long.
  2. Preparation of sgRNA insert
    - Design and obtain oligos containing sgRNA sequence(s) for integration at the BbsI sites in the sgRNA transposon plasmid (Addgene #203359). Sense and anti-sense oligos should contain the appropriate overhang specific to the BbsI cut site to allow for successful ligation (Figure 1). Reconstitute oligos to 100 µM using ultrapure water.
    - Place 4 µL of each of the 100 μM stock of sense and anti-sense oligos along with 4 µL of T4 DNA ligase buffer, 1 µL of PNK, and 27 µL of ultrapure water in a sterile tube and mix well.
    - Phosphorylate and anneal oligos using the following protocol, which can be programmed into a thermocycler:
      37 °C for 30 min
      95 °C for 10 min
      85 °C for 1 min
      75 °C for 1 min
      65 °C for 1 min
      55 °C for 1 min
      45 °C for 1 min
      35 °C for 1 min
      25 °C for 1 min
    - After oligos have been phosphorylated and annealed, dilute 1:50 in ultrapure water and mix well.
  3. Ligation and verification
    - Add 75 ng of purified backbone along with 1 µL of insert (diluted annealed/phosphorylated oligos), 2 µL of T4 DNA ligase buffer, 1 µL of T4 DNA ligase, and water to bring to a final volume of 20 µL, mixing well. Incubate at 16 °C for 1 h.
    - Transform the reaction mixture into chemically competent *E. coli*, such as DH5α strain, spread onto an agar plate supplemented with ampicillin or, alternatively, carbenicillin (100 mg/mL) and incubate overnight at 37 °C [11].
    - Perform a colony PCR using bacterial colonies. We use a primer that binds the U6 promoter (5′-ttcttgggtagtttgcagtttt-3′) as a forward primer and the anti-sense oligonucleotide as a reverse primer. Use the bacterial colony as template DNA, making sure to swirl the colony once in PCR mix before also swirling the same tip in a small volume (i.e., 100 µL) of LB broth for growth of positively identified colonies.
    - Positively identified clones should be grown in overnight cultures in 3–5 mL of LB supplemented with ampicillin (100 μg/mL) on a shaking incubator at 37 °C. After 16 h in culture, plasmid DNA should be extracted from *E. coli* using a Miniprep Plasmid Purification kit.
    - Sanger sequencing can be performed spanning BbsI cut sites to ensure successful assembly of the sgRNA transposon vector. Again, we typically use the U6 primer noted previously for Sanger sequencing. Alternatively, users may also elect to submit samples for full plasmid sequencing service.
- **Maintenance of human embryonic stem cells (hESCs)**
  H9 hESCs were maintained in mTeSR1 on Matrigel-coated plates. Change media daily and passage when needed.
  1. Prepare Matrigel (1 mg/24 mL of DMEM/F12, HEPES).
  2. Coat plates (1.5 mL for each well of a 6-well plate) overnight in 37 °C incubator.
  3. (Optional) Wash cells with 1 mL of gentle cell dissociation buffer. Aspirate.
  4. Incubate with 1 mL of gentle cell dissociation buffer for 3–5 min at room temperature (RT) (check on the microscope for efficient dissociation). Aspirate buffer.
  5. Add fresh mTeSR1, scrape gently with cell lifter, and resuspend the cells with a serological pipette such that they are in small clumps.
  6. Split cells 1:6 (if the well is > 80% confluent) onto 6-well plates for maintenance.
  7. Incubate cells at 37 °C and 5% CO_2_.
- **CRISPRoff transfection**
Figure 2 outlines the experimental procedures in sections E–I.
  **Figure 2.**
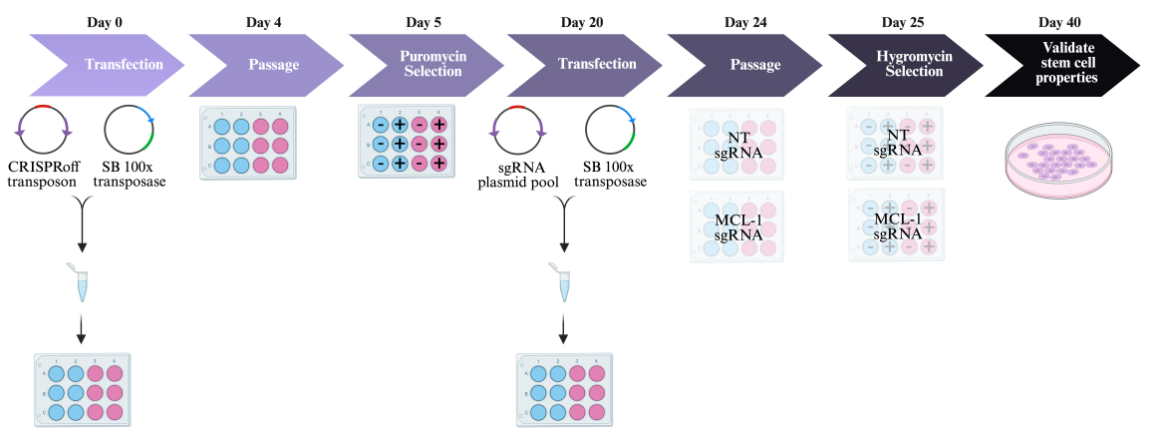
Generation of cell lines. Day 0: Transfect human embryonic stem cells (hESCs) with CRISPRoff transposon and Sleeping Beauty 100× transposase plasmids. Blue wells indicate no DNA negative control and pink wells indicate experimental wells with DNA. Day 4: Passage cells when they reach 70%–80% confluency. Day 5: Start antibiotic selection with puromycin. Blue wells with - indicate no DNA negative control without puromycin. Blue wells with + indicate no DNA negative control with puromycin. Pink wells with - indicate experimental wells with DNA and without puromycin. Pink wells with + indicate experimental wells with DNA and with puromycin. Day 20: Transfect hESCs with sgRNA pool and transpose plasmids. Day 24: Passage cells when they reach 70%–80% confluency. Day 25: Start antibiotic selection with hygromycin. Blue wells with - indicate no DNA negative control without hygromycin. Blue wells with + indicate no DNA negative control with hygromycin. Pink wells with - indicate experimental wells with DNA and without hygromycin. Pink wells with + indicate experimental wells with DNA and with hygromycin. Day 40: Validate stem cell properties. SB = Sleeping Beauty
**Day 1**
  1. Place 250 µL of mTeSR1 containing 1 µL of 10 mM Y27632 into a well of a Matrigel-coated 12-well plate.
  2. Prepare a mixture with 0.75 µg of the Sleeping Beauty transposase plasmid [pCMV(CAT)T7-SB100] and 1.25 µg of the CRISPRoff plasmid in 200 µL of Opti-MEM I reduced serum medium supplemented with 6 µL of TransIT-LT1. Include a mixture without DNA. This is the no-DNA negative control.
  3. After a 15-min incubation at RT, add the transfection mix into a well of a 12-well plate.
  4. Add 1 mL of Accutase per well (for cells in a 6-well plate) to detach hESCs. Incubate for 5 min at 37 °C.
  5. Dilute the Accutase solution by adding 2 mL of DMEM/F12 medium.
  6. Use a cell lifter to remove cells from the well and gently pipette the mixture 2–5 times with the p1000 pipette to fully dissociate the hESC cell colonies to single cells.
  7. Transfer the cells to a conical tube.
  8. Centrifuge the cells at 200× *g* for 4 min at RT.
  9. Resuspend the cell pellet in 1 mL of mTeSR1 containing 10 µM Y27632 and count the cells with a hemocytometer.
  10. Dilute cell suspension to 1.5 × 10^6^ cells/mL.
  11. Place 500 µL of this cell suspension to the well containing the transfection mix.
  12. Incubate cells overnight at 37 °C with 5% CO_2_.
**Day 2**
  1. Aspirate media and replace with fresh mTeSR1 without Y27632.
**Day 4**
  1. Cells should be 70%–80% confluent at this day.
  2. Passage cells into a new 12-well plate as previously outlined in section D.
- **Antibiotic selection**
  1. Ensure that the appropriate puromycin concentration needed has been previously validated from an antibiotic kill curve for the cell type of interest. For the experiments outlined here, a puromycin concentration of 0.8 mg/mL was used for selection.
  2. The day after passaging, supplement mTeSR1 with the appropriate concentration of puromycin.
  3. Continue feeding daily with mTeSR1 supplemented with puromycin for 14 days. It is expected for the negative control cells that did not receive DNA during transfection to die within the first few days. Similarly, untransfected cells in the experimental wells should all die after 14 days. Surviving cells are expected to have integrated the CRISPRoff system into their genome to generate a stable cell line.
  4. From this point forward, media must be supplemented with a half dose of puromycin.
  5. Continue to maintain cells to prepare them for the second transfection.
  6. At this stage, cryopreservation is recommended, as this stable cell line can be used for future transfections with different sgRNA pools.
- **sgRNA transfection**
**Day 1**
  1. Place 250 µL of mTeSR1 containing 1 µL of 10 mM Y27632 into a well of a Matrigel-coated 12-well plate.
  2. Prepare a mixture with 1 µg of the Sleeping Beauty transposase plasmid [pCMV(CAT)T7-SB100] and 1 µg of the sgRNA pool in 200 µL of Opti-MEM I reduced serum medium supplemented with 6 µL of TransIT-LT1.
    - Make sure to include a non-targeting (NT) sgRNA pool in addition to the sgRNA pool targeting the gene of interest.
    - Include a mixture without DNA (DNA negative control).
  3. After a 15-min incubation at RT, add the transfection mix into a well of a 12-well plate.
  4. Add 1 mL of Accutase per well (for cells in a 6-well plate) to detach hESCs. Incubate for 5 min at 37 °C.
  5. Dilute the Accutase solution by adding 2 mL of DMEM/F12 medium.
  6. Use a cell lifter to remove cells from well and gently pipette the mixture 2–5 times with the p1000 pipette to fully dissociate the hESC cell colonies to single cells.
  7. Transfer the cells in a conical tube.
  8. Centrifuge the cells at 200× *g* for 4 min at RT.
  9. Resuspend the cell pellet in 1 mL of mTeSR1 containing 10 µM Y27632 and count the cells with a hemocytometer.
  10. Dilute cell suspension to 1.5 × 10^6 ^cells/mL.
  11. Place 500 µL of this cell suspension to the well containing the transfection mix.
  12. Incubate cells overnight at 37 °C with 5% CO_2_.
**Day 2**
  1. Aspirate media and replace with fresh mTeSR1 without Y27632.
**Day 4**
  1. Cells should be 70%–80% confluent at this day.
  2. Passage cells into a new 12-well plate as previously outlined in section D.
- **Selection**
  1. Ensure that the appropriate hygromycin B concentration needed is determined from an antibiotic kill curve for the specific cell type of interest. A hygromycin B concentration of 60 mg/mL was used for selection in our experiments.
  2. The day after passaging, supplement mTeSR1 with the appropriate concentration of hygromycin.
  3. Continue feeding daily with hygromycin supplementation for 14 days. It is expected for the negative control cells that did not receive DNA during transfection to die within the first few days. Similarly, un-transfected cells in the experimental wells should all die after 14 days. The sgRNA transposons are expected to have integrated the chromosomes of surviving cells, giving rise to a stable cell line.
  4. From this point forward, media must be supplemented with a half dose of puromycin and hygromycin B.
- **Validating pluripotency and genome integrity (Figure 3A and 3B)**
  **Figure 3.**
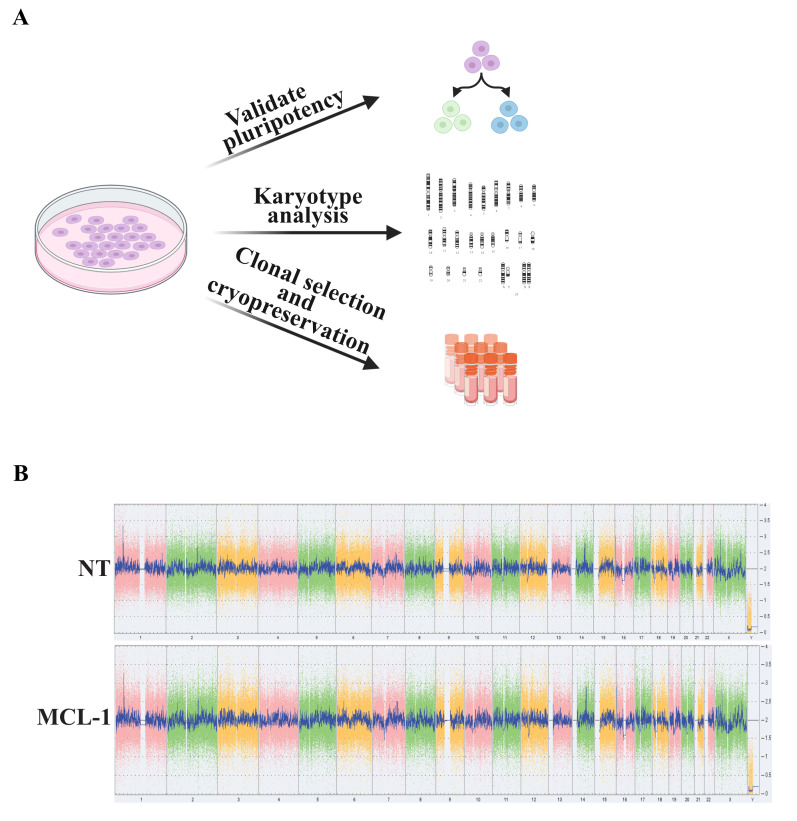
Validation of stem cell pluripotency and genome integrity. (A) Validate stem cell properties by testing pluripotency, karyotyping, and by cryopreserving selected stem cell colonies. (B) Confirm genomic stability of NT and MCL-1 cells using the KaryoStat+ Assay (Thermo Fisher Scientific). No aberrations were detected in cells assessed for the studies presented here.
  1. Perform colony hESC selection and continue to maintain cells.
  2. Once clonal lines have been stabilized, use cells to test pluripotency.
  3. Karyotype cells to validate genomic integrity of the clones. KaryoStat+ Assay Service from Thermo Fisher was used for the cell lines presented here.
  4. Cryopreserve cells for future experiments.
- **Validating knockdown of embryonic lethal genes**
  As previously discussed, silencing of MCL-1 in stem cells is lethal. Cells were treated with Q-VD-OPh (QVD), a pan-caspase inhibitor, to prevent cell death when MCL-1 is silenced with doxycycline (DOX) treatment. Passage MCL-1 and NT cell lines onto 6-well Matrigel-coated plates. Set up plates to have four experimental groups: -DOX, +DOX, +DOX +QVD, and +DOX +DMSO (QVD vehicle control). Once cells reach 40%–50% confluency, proceed to steps below.
**Day 0**
  1. Supplement mTeSR1 and antibiotic cocktail with 1 µg/mL of DOX and/or 25 µM of QVD and add to the corresponding wells.
**Days 1–2**
  1. Change media daily and continue to add DOX and/or QVD for a total of 72 h.
**Day 3**
  1. Once treatment reaches 72 h, collect cells for experimental endpoint assay (e.g., quantitative RT-PCR to measure gene expression of MCL-1).
  2. Wash cells once with 1× PBS.
  3. Add 500 µL of TRIzol reagent. Note: If using TRIzol, make sure to collect cells in a chemical fume hood.
  4. Scrape cells with a cell lifter and collect in a 1.5 mL centrifuge tube. Samples can be stored at -80 °C or you may proceed with RNA isolation.
- **RNA isolation**
  This protocol was adapted from Invitrogen TRIzol reagent protocol. All steps using TRIzol should be done in the chemical fume hood.
  1. Make sure to perform RNA isolation in a RNase-free area. Spray gloves, pipettes, pipette tip boxes, tubes, tube racks, and bench with RNaseZAP.
  2. If samples were previously frozen, allow them to incubate at RT for at least 5 min.
  3. Add 100 µL of chloroform to sample.
  4. Shake tubes vigorously for ~15 s.
  5. Incubate the samples at RT for 2–3 min.
  6. Centrifuge the samples at 12,000× *g* for 15 min at 4 °C.
  7. Remove the aqueous phase of the sample and place in a new tube.
*Note: Make sure to dispose of TRIzol and other hazard chemicals according to institutional policies.*
  8. Add 250 µL of 2-Propanol to the aqueous phase to precipitate RNA.
  9. Gently invert ~10 times.
  10. Incubate at RT for 25 min.
  11. Centrifuge at 12,000× *g* for 10 min at 4 °C. Keep samples on ice from this step onwards.
  12. Remove the supernatant from the tube, leaving only the RNA pellet.
  13. Wash the pellet with 500 μL of 75% ethanol.
  14. Vortex the sample briefly and then centrifuge the tube at 10,000× *g* for 10 min at 4 °C.
  15. Pipette away as much of the wash as possible and allow the RNA pellet to semi-dry.
  16. Resuspend pellet in 30 μL of DEPC-treated water. Store at -80 °C.
- **DNase treatment of RNA**
  This protocol was adapted from New England Biolabs DNase I Reaction protocol.
  1. Set up DNase I reaction on ice (Recipe 1). The volumes indicated on the Recipe are per sample.
  2. Incubate in the thermocycler at 37 °C for 10 min.
  3. Inactivate the DNase I by adding 0.5 µL of 0.1 M EDTA solution to the reaction mixture.
  4. Heat in the thermocycler for 10 min at 75 °C.
  5. The RNA sample is now ready to be used for reverse transcription.
- **cDNA synthesis and quantitative RT-PCR**
  This protocol was adapted from Thermo Fisher High-Capacity cDNA Reverse Transcription kit and SYBR Green reagent.
  1. Set reverse transcription master mix (Recipe 2). The volumes indicated on the Recipe are per sample.
  2. Add 10 µL of the master mix to 10 µL of RNA sample (2 µg) in a PCR tube and mix well by vortexing. Briefly spin down tubes.
  3. Run samples in the thermal cycler using the following conditions:
    25 °C for 10 min
    37 °C for 120 min
    85 °C for 5 min
    4 °C hold
  4. Final cDNA reaction is 20 µL. Dilute to 200 µL by adding 180 µL of nuclease-free H_2_O after reaction. Studies here involved 5 µL of cDNA for qPCR.
  5. QuantStudio 3 Real-Time PCR machine, SYBR Green master mix, and manufacturer’s instructions were used to set up the assay. The primers used to measure gene expression of *MCL1* are found in Table S2. Figure 4 contains results of the experimental validation in hESCs.
    **Figure 4.**
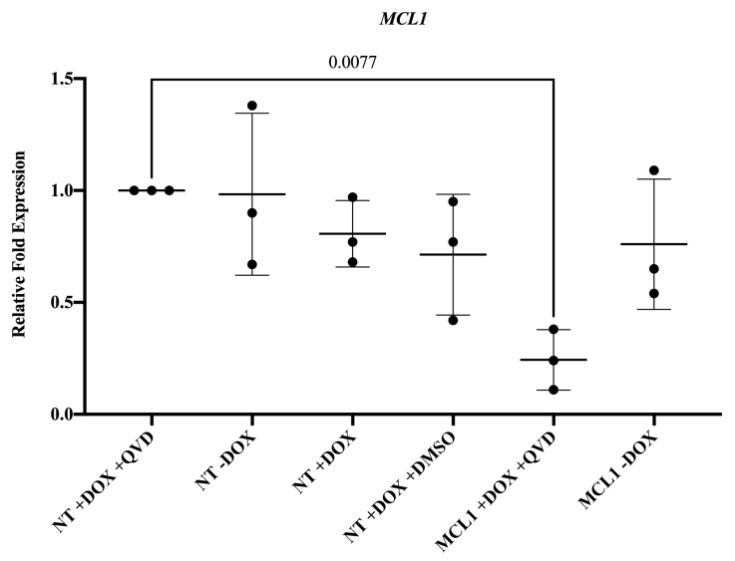
Silencing *MCL-1* in human embryonic stem cells (hESCs). Gene expression of *MCL-1* is significantly downregulated in MCL-1 hESCs with doxycycline and Q-VD-OPh treatment. Normalized to control (NT +DOX +QVD). Statistical test: ordinary one-way ANOVA with Dunnett’s multiple comparisons test. DOX = doxycycline. QVD = Q-VD-Oph.
- **Differentiation**
  This is a step-by-step protocol that was adapted from Douvaras and Fossati (2015) [8].
**Day -3: Replating hESCs**
  1. When the cells reach 70%–90% confluency, remove the medium and add 1 mL of Accutase.
  2. Incubate the plate in a 37 °C incubator for 5 min.
  3. Dilute the Accutase solution by adding 2 mL of DMEM/F12 medium.
  4. Use a cell lifter to remove cells from the well and gently pipette the mixture 2–5 times with the p1000 pipette to fully dissociate the hESC cell colonies to single cells.
  5. Transfer the cells to a conical tube.
  6. Centrifuge the cells at 200× *g* for 4 min at RT.
  7. Resuspend the cell pellet in 1 mL of mTeSR1 containing 10 µM Y27632 and count the cells with a hemocytometer.
  8. Plate 8 × 10^4^ cells per well on a Matrigel-coated 6-well plate.
**Day -2: Maintaining hESCs**
  1. Aspirate and add fresh mTeSR1 media to remove Y27632.
**Day -1: Maintaining hESCs**
  1. Continue to maintain cells in mTeSR1.
**Day 0: Neural induction**
  1. By now, colonies should have grown evenly and reached 80% confluency. When cells have reached this point, proceed with differentiation.
  2. Aspirate medium and add NIM to induce differentiation.
  3. Change NIM daily until cells have reached day 8.
**Day 8: Oligodendrocyte lineage induction**
  1. Aspirate medium and add N2 medium to direct cells to oligodendrocyte lineage commitment.
**Day 9: Replate neural precursor cells**
  1. Remove the medium and add 1 mL of Accutase.
  2. Incubate the plate in a 37 °C incubator for 5 min.
  3. Dilute the Accutase solution by adding 2 mL of DMEM/F12 medium.
  4. Use a cell lifter to remove cells from the well and gently pipette the mixture 2–5 times with the p1000 pipette to fully dissociate cells.
  5. Transfer the cells to a conical tube.
  6. Centrifuge the cells at 200× *g* for 4 min at RT.
  7. Resuspend the cell pellet in 1 mL of N2 medium containing 10 µM Y27632 and count the cells with a hemocytometer.
  8. Plate 1.5 × 10^6^ cells per well on a Matrigel-coated 6-well plate. Use Matrigel-coated 35 mm glass-bottom plates for immunofluorescence. Supplement medium with 10 µM Y27632.
**Day 10: Doxycycline administration to inducibly silence MCL-1**
  1. Aspirate medium and add N2 medium supplemented with 1 µg/mL doxycycline.
  2. Change N2 medium supplemented with 1 μg/mL doxycycline every 24 h.
**Day 14: Collect cells for western blot, quantitative RT-PCR, and immunofluorescence**
- **Cell lysis and protein extraction for western blotting**
  1. Prepare lysis buffer on ice. Make enough to use 100 mL per well of a 6-well plate.
  2. Aspirate media from wells.
  3. Wash once with 1× PBS.
  4. Add 100 µL of lysis buffer to each well.
  5. Scrape wells vigorously with cell lifter and transfer sample to a 1.5 µL centrifuge tube.
  6. Place tubes on ice for 30 min. Vortex every 10 min.
  7. Spin down samples at 14,000× *g* for 30 min.
  8. Collect supernatant and transfer to a new 1.5 µL centrifuge tube.
  9. Store sample at -20 °C.
- **Measuring protein expression by western blot**
  1. Prepare SDS for Tris-Gly-SDS buffer.
    - Dissolve 200 g of SDS in 900 mL of ddH_2_O.
    - Heat to 68 °C and stir with a magnetic stirrer to assist dissolution.
    - If necessary, adjust the pH to 7.2 by adding a few drops of concentrated HCl.
    - Adjust the volume to 1 L with ddH_2_O.
  2. Determine protein concentration using the Thermo Scientific BCA Protein Assay kit.
  3. Dilute 30 µg of protein in 1% Triton and LDS with 2-Mercaptoethanol.
  4. Incubate samples at 95 °C for 5 min.
  5. Run samples on a 10-well 4%–20% Mini-Protean TGX precast protein gel in Tris-Gly-SDS buffer.
  6. Transfer gel onto polyvinylidene difluoride membranes at 4 °C overnight.
  7. Block membrane in 5% milk diluted in TBST for one hour at RT on a benchtop rocker.
  8. Incubate with primary antibody overnight on a benchtop rocker. The antibodies used here can be found in Table S3.
  9. Wash membrane with TBST for 5 min on a benchtop rocker. Repeat two more times.
  10. Incubate with HRP-conjugated secondary antibodies against mouse or rabbit IgG for 1 h at RT on a benchtop rocker.
  11. Wash membrane with TBST for 5 min on a benchtop rocker. Repeat two more times.
  12. Develop blots with ECL Plus reagent and image on the Chemiluminescent Imager.
  13. Bands were quantified with Image Studio Lite. Figure 5A and 5B contain the results of the experimental validation in committed oligodendrocyte neural precursor cells.
    **Figure 5.**
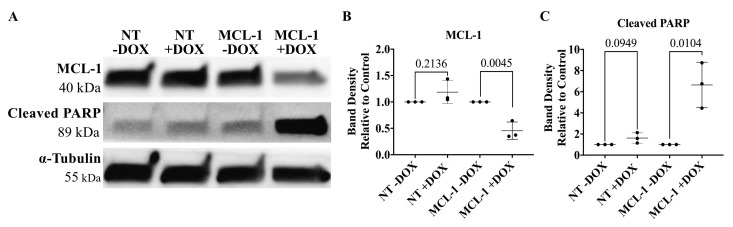
Silencing MCL-1 in oligodendrocyte neural precursor cells. (A) Representative image of MCL-1 protein expression and cleaved PARP protein expression in committed oligodendrocyte neural precursor cells following 96 h of doxycycline treatment in NT and MCL-1 CRISPRoff cell lines. (B) MCL-1 is significantly downregulated in committed oligodendrocyte neural precursor cells following 96 h doxycycline treatment in MCL-1 cell line. Normalized to respective controls (NT -DOX and MCL-1-DOX). Statistical test: Student’s *t*-tests. (C) Cleaved PARP is significantly upregulated in oligodendrocyte neural precursor cells following 96 h doxycycline treatment in MCL-1 cell line. Normalized to respective controls (NT -DOX and MCL-1 -DOX). Statistical test: multiple *t*-tests. DOX = doxycycline. QVD = Q-VD-OPh.
- **Immunofluorescence**
  1. Fix cells with 4% PFA in 1× PBS for 20 min at RT.
  2. Permeabilize cells with 1% Triton for 10 min at RT.
  3. Block with 10% BSA prepared in 1× PBS for 1 h at RT.
  4. Incubate with primary antibody diluted in 10% BSA overnight at 4 °C. The antibodies used here can be found in Table S3.
  5. Wash with 1× PBS. Repeat two more times.
  6. Incubate with secondary antibody diluted in 10% BSA at RT for 1 h.
  7. Wash with 1× PBS. Repeat two more times.
  8. Incubate with Hoechst for 10 min.
  9. Wash with 1× PBS.
  10. Mount with Fluoromount-G slide mounting medium.
  11. Image cells on a confocal microscope. Silencing MCL-1 in committed oligodendrocyte neural precursor cells leads to changes in expression of OLIG2 and NKX2.2 (Figure 6).
    **Figure 6.**
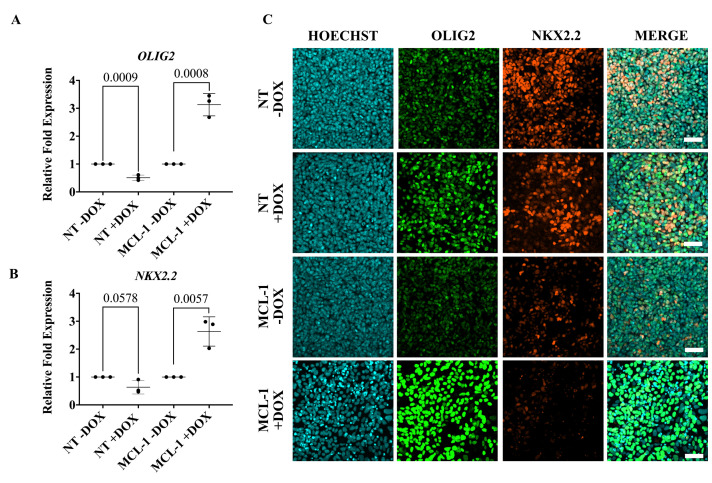
Dysregulation of transcription factors that modulate oligodendrocyte lineage commitment following MCL-1 suppression. (A) Gene expression of *OLIG2* was significantly increased in committed OL neural precursor cells (NPCs) following 96 h doxycycline treatment in MCL-1 cell line. Normalized to respective controls (NT -DOX and MCL-1 -DOX). Statistical test: multiple t-tests. (B) Gene expression of *NKX2.2* was significantly increased in committed OL NPCs following 96 h doxycycline treatment in MCL-1 cell line. Normalized to respective controls (NT -DOX and MCL-1 -DOX). Statistical test: multiple t-tests. (C) Silencing of MCL-1 upregulates protein expression of OLIG2 and downregulates NKX2.2 in committed OL NPCs. DOX = doxycycline. Scale bar = 50 µm.

## Data analysis

Detailed description of analysis can be found in the figure legends.

## Validation of protocol

We validated the use of this protocol by silencing MCL-1, an anti-apoptotic protein, at an early state of oligodendrocyte differentiation in vitro. MCL-1 is required for the survival of hPSCs in vitro and thus needs to be silenced after the initiation of the differentiation process. All experiments presented have been replicated using three or more technical replicates and at least three biological replicates. Controls included NT sgRNAs.

## General notes and troubleshooting

**General notes**

1. The maintenance of the stem cells should be rigorously tested to assure they are pluripotent at the start of the protocol.
2. Dose response of the selection drugs is essential to increase the efficiency of silencing.
3. Oligodendrocyte markers and primers should be rigorously validated.

## Supplementary information

The following supporting information can be downloaded here:

1. Table S1. MCL-1 and NT sgRNAs
2. Table S2. MCL-1 primer sequences
3. Table S3. Antibodies
